# Molecular Mechanisms Underlying Anti-Inflammatory Actions of 6-(Methylsulfinyl)hexyl Isothiocyanate Derived from Wasabi (*Wasabia japonica*)

**DOI:** 10.1155/2012/614046

**Published:** 2012-08-15

**Authors:** Takuhiro Uto, De-Xing Hou, Osamu Morinaga, Yukihiro Shoyama

**Affiliations:** ^1^Department of Pharmacognosy, Faculty of Pharmaceutical Sciences, Nagasaki International University, 2825-7 Huis Ten Bosch, Sasebo, Nagasaki 859-3298, Japan; ^2^Course of Biological Science and Technology, United Graduate School of Agricultural Sciences, Kagoshima University, Korimoto 1-21-24, Kagoshima 890-0065, Japan

## Abstract

6-(Methylsulfinyl)hexyl isothiocyanate (6-MSITC) is a major bioactive compound in wasabi (*Wasabia japonica*), which is a typical Japanese pungent spice. Recently, *in vivo* and *in vitro* studies demonstrated that 6-MSITC has several biological properties, including anti-inflammatory, antimicrobial, antiplatelet, and anticancer effects. We previously reported that 6-MSITC strongly suppresses cyclooxygenase-2 (COX-2), inducible nitric oxide synthase (iNOS), and cytokines, which are important factors that mediate inflammatory processes. Moreover, molecular analysis demonstrated that 6-MSITC blocks the expressions of these factors by suppressing multiple signal transduction pathways to attenuate the activation of transcriptional factors. Structure-activity relationships of 6-MSITC and its analogues containing an isothiocyanate group revealed that methylsulfinyl group and the length of alkyl chain of 6-MSITC might be related to high inhibitory potency. In this paper, we review the anti-inflammatory properties of 6-MSITC and discuss potential molecular mechanisms focusing on inflammatory responses by macrophages.

## 1. Introduction

Isothiocyanates (ITCs) are a group of naturally occurring sulfur compounds containing –N=C=S functional group, available often abundantly from many cruciferous vegetables. ITCs are stored as glucosinolate precursors in the plants. The damage of plant tissue such as chopping and mastication activates myrosinase which hydrolyses the glucosinolate (myrosinase-glucosinolate system), and the resultant ITCs play a key role in the defense against herbivores and pathogens [1, 2]. There are a significant number of naturally occurring and synthetic ITCs, and numerous studies have demonstrated the chemopreventive and anti-inflammatory properties of ITCs *in vitro* and *in vivo* [3–5]. Accumulating evidence suggests that ITCs exert their effects through a variety of signaling pathways involved in detoxification, inflammation, apoptosis, and cell cycle regulation, among others [4–6].

**Figure 1:**
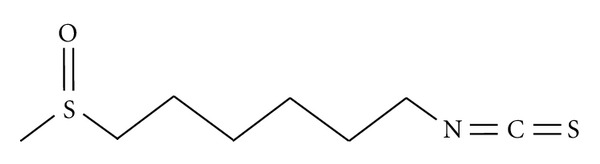
Chemical structure of 6-MSITC. 6-MSITC contains methyl sulfoxide group and ITC group linked by alkyl chain.

Wasabi (*Wasabia japonica*) is a member of the Brassicaceae family of vegetables, and its rhizome is a very popular pungent spice in Japan. Several studies have shown that wasabi has multiple physiological functions, such as appetite enhancement [7], antimicrobial activity [8], inhibition of platelet aggregation [9], and the suppression of *N*-methyl-*N*′-nitro-*N*-nitrosoguanidine-induced rat gastric carcinogenesis [10]. Wasabi differs from other Brassicaceae species in that it contains higher concentration of ITCs, especially long-chain ITCs. The bioactive components of wasabi have been identified as a series of ITC analogues, of which 6-(methylsulfinyl)hexyl isothiocyanate (6-MSITC or 6-MITC) (Figure 1) is a major active compound in wasabi. Several lines of evidence demonstrated the pharmacological potencies of 6-MSITC, such as anti-inflammatory [11–13], antimicrobial [14], antiplatelet [15], and anticancer [16–18] effects. We previously reported that 6-MSITC strongly suppresses inflammatory mediators by regulating the signaling pathways [11–13]. In this paper, we describe the anti-inflammatory properties of 6-MSITC and discuss potential molecular mechanisms with special attention to several inflammatory factors in macrophages.

## 2. Chemistry and Extraction of 6-MSITC

A number of analogues of ITCs are isolated from wasabi, and the main pungent compound of wasabi is allyl ITC. Ina et al. [19] and Etoh et al. [20] reported that the characteristic flavor of wasabi depends on the methylthioalkyl ITCs and methylsulfinylalkyl ITCs. The methylthioalkyl ITCs are found in wasabi alone, whereas methylsulfinylalkyl ITCs are found in both wasabi and horseradish. Wasabi contains much higher levels of all methylsulfinylalkyl ITCs than horseradish [19]. One of the methylsulfinylalkyl ITCs is 6-MSITC, and the chemical structure of 6-MSITC contains methyl sulfoxide group and ITC group linked by alkyl chain (Figure 1). Although the ITCs are generated throughout the entire plant of wasabi, the root is the predominant site of storage. Hara et al. [21] found the myrosinase-glucosinolate composition of wasabi is in the epidermis and vascular cambium of the root. Indeed, wasabi root is particularly rich in most ITCs [19]. 

Several groups isolated 6-MSITC from wasabi root by monitoring the cellular bioactivities. Ono et al. [22] purified 6-MSITC from the water-soluble fractions of wasabi by monitoring the growth inhibition of MKN-28 cells. The water-soluble fraction of wasabi roots was fractionated by Sephadex G-15 gel filtration and reverse-phase HPLC successfully, and active compound was finally collected by preparative HPLC. The spectroscopic data, including fast atom bombardment mass spectrometry (FAB-MS) and electron ionization mass spectrometry (EI-MS), determined a molecular ion peak at [M]^+^ *m/z* 205 and the molecular formula of C_8_H_15_NOS_2_. Furthermore, IR and NMR completely confirmed that an active compound is 6-MSITC. Morimitsu et al. [23] extracted the smashed wasabi root to give an active ethyl acetate fraction, resulting in 6-MSITC as the major active compound of glutathione *S-*transferase (GST) activity after purification by silica gel column chromatography and preparative HPLC. The content of 6-MSITC in wasabi is ~550–556 *μ*g/g wet body weight of wasabi root.

## 3. Effect of 6-MSITC on Inflammatory Factors

Inflammation is one of the most important host defense systems against tissue injuries and pathogen invasion [24]. In the inflammatory process, macrophages play a central role in induction of inflammatory enzymes, cytokines, chemokines, and other inflammatory factors. Overexpression of these inflammatory factors by macrophages has been implicated in the pathophysiology of many inflammatory diseases, such as rheumatoid arthritis, atherosclerosis, chronic hepatitis, pulmonary fibrosis, and inflammatory brain diseases [25, 26]. Lipopolysaccharide (LPS), a component of Gram-negative bacterial cell wall, activates macrophages to produce prostaglandin E_2_ (PGE_2_) by cyclooxygenase-2 (COX-2), nitric oxide (NO) by inducible NO synthase (iNOS), and inflammatory cytokines through the activating multiple signaling pathways [27, 28]. Thus, the biological reduction of LPS-inducible inflammatory factors is considered to be an effective strategy for inflammatory diseases. 

### 3.1. COX-2

COXs catalyze the synthesis of prostaglandins from arachidonic acid. There are two isoforms of COX, designated COX-1 and COX-2, which are encoded by different genes. COX-1 is constitutively expressed in most tissues and believed to be responsible for normal physiological functions [29]. In contrast, COX-2 is not detectable in normal tissues or resting immune cells, but it could be induced by LPS, inflammatory cytokines, growth factors, and carcinogens [30, 31]. 

6-MSITC suppressed LPS-induced COX-2 expression and PGE_2_ release in murine macrophage cell lines RAW264 and human U937 monocytic cells without affecting the constitutive COX-1 expression [11]. Molecular analysis demonstrated that 6-MSITC blocked LPS-induced COX-2 expression in transcriptional level. In the COX-2 gene, *cis*-acting elements including nuclear factor *κ*B (NF-*κ*B), CCAAT/enhancer-binding protein (C/EBP), and cyclic AMP-response element (CRE) have been identified to play a critical role in regulating transcription [32–36]. Moreover, single site of NF-*κ*B, C/EBP, or CRE cannot sufficiently respond to induce COX-2 transcription activity, and two of these *cis*-acting elements are at least recruited to achieve maximal induction of transcription [32]. 6-MSITC inhibited LPS-induced COX-2 expression by suppressing transcriptional factors binding to the first 327 base pairs in the 5′ flanking regions of COX-2 gene [11]. Moreover, mutation of a single NF-*κ*B, C/EBP, or CRE promoter element did not abrogate the effect of 6-MSITC. Thus, the inhibition of at least two of these *cis*-elements is required to achieve the maximal inhibitory action of 6-MSITC on COX-2 gene expression, suggesting that the inhibitory effect of 6-MSITC on COX-2 expression could be obtained by targeting the signaling pathways leading to at least two promoter elements including NF-*κ*B, C/EBP, and CRE sites.

In mouse RAW264 macrophages, COX-2 expression was activated by interferon (IFN)-*γ* and 12-*O*-tetradecanonoylphorbol-13-acetate (TPA) in the same manner as LPS [13]. Interestingly, 6-MSITC downregulated COX-2 expression induced by LPS and IFN-*γ* but did not suppress that induced by TPA. These data indicated that LPS, IFN-*γ*, and TPA regulate COX-2 expression through different pathways, and 6-MSITC acts as a potent inhibitor against LPS- or IFN-*γ*-induced COX-2 expression.

### 3.2. iNOS

NO is produced endogenously during arginine metabolism by isoforms of NOS [37, 38]. NO has a number of important biological functions, including tumor cell killing, host defense against intracellular pathogens, neurotransmission, and inhibition of platelet aggregation [39]. However, excess NO is a potent mediator and regulator of inflammatory responses [40, 41] and also has a multifaceted role in process of cancer [42]. NO is synthesized from L-arginine by NOS, which exists as three distinct isoforms of NOSs, including endothelial nitric oxide synthase (eNOS), neuronal nitric oxide synthase (nNOS), and iNOS [43]. iNOS is induced by various inflammatory stimuli such as LPS and inflammatory cytokines in macrophages, hepatocytes, and endothelial cells [44–46]. A large amount of NO catalyzed by iNOS plays a key role in the various forms of inflammation and carcinogenesis [46–48]. 

Noshita et al. [49] investigated the inhibitory activities of 6-MSITC and other synthesized ITCs against LPS-induced NO production using mouse peritoneal macrophages and mouse J774.1 macrophage-like cells. Among the tested ITCs, 6-MSITC indicated the strongest inhibition of NO production. We also reported that 6-MSITC reduced NO production, and this inhibition depends on the suppression of iNOS expression at the transcriptional level as well as COX-2 expression [12]. 

### 3.3. Inflammatory Cytokines/Chemokines

Inflammatory cytokines such as interleukin (IL), interferon (IF), and tumor necrosis factor (TNF) play important roles in the regulation of the immune system [50]. Similar to PGE_2_ and NO, overproduction of inflammatory cytokines from macrophages causes oxidative stress, systemic inflammation, and cell dysfunction. In addition, chemokines, which are chemotactic cytokines, are well known as multifunctional mediators of gene transcription, cell proliferation, and leukocyte recruitment to inflamed tissues [50]. Chen et al. [51] performed gene expression profiling by DNA microarray in macrophages. Among a total of 22,050 gene probes, LPS upregulated the expression level of 406 genes (1.8% of the total gene probes) and downregulated 717 genes (3.2% of the total genes probes) by ≥3-fold. The number of genes affected by 6-MSITC consisted of 58% of downregulated genes by LPS and 47% of upregulated genes by LPS. Gene ontology analysis revealed that the gene groups highly affected by 6-MSITC were associated with “inflammatory responses, signal transduction, cytokine activities, hydrolase activity, kinase activity, receptor activity, transferase activity, nucleic acid binding and apoptosis.” According to gene profiling and real-time PCR for further confirmation, the upregulation of inflammatory cytokine genes, such as IL-1*β*, IL-6, and TNF, by LPS was reduced by 6-MSITC. 6-MSITC attenuated the expression of IF-inducible genes (IFI1 and IFI47), which are involved in IF-mediated cell proliferation and differentiation. The inductions of IL receptors (IL10ra, IL23ra, and IL4ra) by LPS were also reduced by 6-MSITC. These results suggested 6-MSITC inhibition of various inflammatory genes may explain its strong anti-inflammatory effects. On the other hand, 6-MSITC restored the expression levels of LPS-reduced CC chemokines (CCL11 and CCL25), IL-3, and receptors (IL1ra12, IL8ra, TNFRSF23, and TNFRSF4) to control levels. Overall, these data suggest that 6-MSITC might regulate the expression of inflammatory and anti-inflammatory cytokines.

## 4. Effect of 6-MSITC on Transcriptional ****Regulation Involved in Inflammatory Factors

### 4.1. Mitogen-Activated Protein Kinase (MAPK)

MAPK signaling pathways play a critical role in the regulation of inflammatory response and coordinate the induction of many genes encoding inflammatory factors [52–54]. MAPK has three major subfamily members including extracellular-regulated protein kinase (ERK), p38 kinase, and c-Jun NH_2_-protein kinase (JNK). The activated form of each MAPK phosphorylates and activates other kinases or transcriptional factors, thereby altering the expression of the target genes such as COX-2, iNOS, and inflammatory cytokines [3, 34, 54]. Our data demonstrated that 6-MSITC blocked LPS-induced phosphorylation of all MAPKs and MAPK kinases (MAPKKs) [11]. Furthermore, MAPK-specific inhibitors (U0126 for MEK1/2, SB203580 for p38 kinase, and SP600125 for JNK) demonstrated that LPS-induced COX-2 expression was partially suppressed by the treatment with single inhibitor. However, the combination treatment of two inhibitors markedly reduced COX-2 expression. In particular, cotreatment with three inhibitors completely inhibited COX-2 expression. Thus, these data indicated that three MAPK pathways cooperatively activated COX-2 expression, and 6-MSITC attenuated COX-2 expression by blocking all of three MAPK pathways. On the other hand, only JNK-specific inhibitor SP600125 suppressed LPS-induced iNOS expression, while ERK-specific inhibitor U0126 and p38-specific inhibitor SB203580 did not, suggesting that only JNK pathway required iNOS expression, and 6-MSITC might suppress iNOS expression by blocking JNK phosphorylation.

### 4.2. Activator Protein-1 (AP-1)

**Figure 2:**
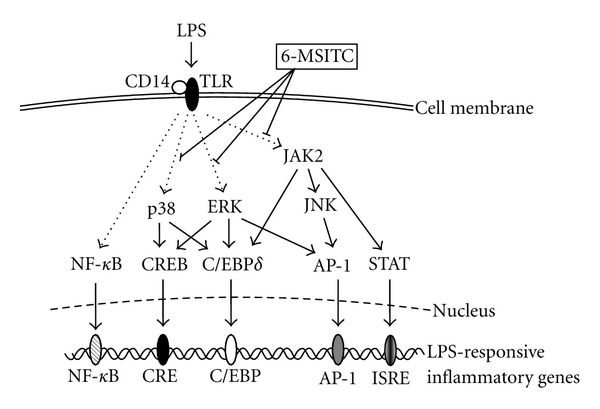
Schematic molecular model of 6-MSITC on the suppression of LPS-induced inflammatory factors.

AP-1, a heterodimer of Jun (c-Jun, Jun B, and JunD) and Fos (cFos, Fos B, Fra-1, and Fra-2), plays an important role in inflammatory responses [54, 55]. AP-1 is minimally activated under normal physiologic conditions, but is dramatically activated by inflammatory stimuli, like LPS [55]. The activated AP-1 binds to the promoter elements, which regulate the transcription of inflammatory genes such as COX-2, iNOS, TNF*-*α**, IL-1*β*, and IL-6 [55]. 6-MSITC completely inhibited LPS-induced phosphorylation of c-Jun, which is a major component of AP-1 in c-Jun/c-Fos heterodimer form [11, 12]. MAPK inhibitors revealed that ERK and JNK signaling pathways cooperatively regulate COX-2 expression by activating AP-1 because SP600125 and U0126, but not SB203580, inhibited c-Jun phosphorylation [11]. Moreover, SP600125 suppressed c-Jun phosphorylation and iNOS expression, suggesting that 6-MSITC might inhibit iNOS expression by blocking JNK-mediated AP-1 activation (Figure 2) [12].

### 4.3. CREB and C/EBP

The promoter region of COX-2 gene contains binding sites for CREB and C/EBP. LPS-induced phosphorylation of CREB and nuclear translocation of C/EBP can regulate COX-2 gene expression through CRE site and C/EBP site, respectively [56–58]. Several lines of studies have shown that the binding of CREB to CRE site depends on the phosphorylation of CREB [33, 59, 60], and the binding of C/EBP to COX-2 promoter is preceded by nuclear translocation of C/EBP [28, 56, 61]. 6-MSITC inhibited LPS-induced phosphorylation of CREB [11]. Moreover, LPS-induced expression and nuclear translocation of C/EBP*δ*, but not C/EBP*β*, were blocked by 6-MSITC [11]. The analysis by MAPK inhibitors demonstrated that ERK and p38 kinase pathways cooperatively regulate COX-2 expression by activating CREB and C/EBP*δ* because ERK-specific inhibitor U0126 and p38 kinase-specific inhibitor SB203580 suppressed CREB phosphorylation and C/EBP*δ* expression, but JNK-specific inhibitor SP600125 did not. Therefore, 6-MSITC blocked LPS-induced COX-2 expression by suppressing ERK and p38 kinase signaling cascades leading to the activation of CREB and C/EBP*δ* (Figure 2). The promoter region of iNOS gene also contains binding site for C/EBP. Although 6-MSITC inhibited ERK and p38 kinase signaling cascades leading to C/EBP*δ*, U0126 and SB203580 had no influence on LPS-induced iNOS expression [12]. Hecker et al. reported that C/EBP*β* may involve iNOS gene expression synergistically with NF-*κ*B in primary rat hepatocytes [62]. Our data showed that LPS had no influence on the nuclear translocation of C/EBP*β* [11]. These data suggest that LPS-induced iNOS expression, which was reduced by 6-MSITC, was not involved in C/EBP binding site of iNOS gene promoter.

### 4.4. NF-*κ*B

NF-*κ*B is involved in the induction of inflammatory genes and activated by the inflammatory responses during viral and bacterial infections [63, 64]. Previous analysis has demonstrated that a number of natural occurring compounds suppressed LPS-induced expression of COX-2, iNOS, and inflammatory cytokines by blocking degradation of inhibitor *κ*B (I*κ*B)-*α* in mouse macrophage cells [56, 65, 66]. However, 6-MSITC had no influence on phosphorylation and degradation of I*κ*B-*α* and nuclear translocation of p65 [11, 12]. Thus, 6-MSITC may inhibit inflammatory factors without the suppression of I*κ*B degradation.

### 4.5. Janus Kinase- (JAK-) Signal Transducers and Activators of Transcription (STAT)

The JAK-STAT pathway is an important inflammatory signaling pathway. JAK family, a protein tyrosine kinase (PTK), contains four members, JAK1, JAK2, JAK3, and tyrosine kinase 2 (TYK2), which are differentially regulated in response to various cytokines [67]. Binding of ligands to its receptors activates the phosphorylation of JAK, which subsequently leads to STAT phosphorylation. Phosphorylated STATs translocate to nuclear and regulate the transcription of target genes such as iNOS and COX-2 and inflammatory cytokines/chemokines [68–70]. Our data demonstrated that AG490 (JAK2-specific inhibitor) abolished LPS-induced expression of COX-2 (unpublished data) and iNOS [12]. Furthermore, AG490 reduced LPS-induced c-Jun phosphorylation, a major component of AP-1, and C/EBP*δ* activation. Molecular analysis with AG490 and SP600125 demonstrated that JAK2 acts upstream of JNK leading to AP-1 activation, and JNK cannot regulate the C/EBP*δ* activation. Moreover, 6-MSITC blocked LPS-induced JAK2 phosphorylation and its downstream pathways. Taken together, JAK2 might upregulate the expression of inflammatory factors through the induction of STAT phosphorylation, C/EBP*δ* expression, and JNK-mediated AP-1 activation. Moreover, 6-MSITC suppresses LPS-induced JAK2 phosphorylation leading to the induction of inflammatory factors (Figure 2).

## 5. Structure-Activity**  **Relationship

**Figure 3:**
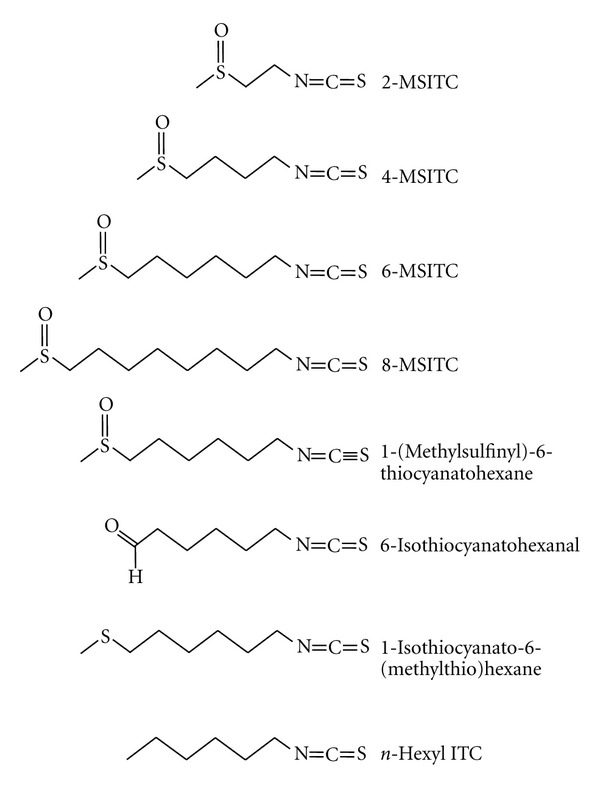
Chemical structures of analogues of 6-MSITC. 4-MSITC (sulforaphane), 2-MSITC, and 8-MSITC are analogues of 6-MSITC containing the different length of alkyl chain. 1-(Methylsulfinyl)-6-thiocyanatohexane substituted ITC group in 6-MSITC with thiocyanate group. 6-Isothiocyanatohexanal, 1-isothiocyanato-6-(methylthio)hexane, and *n*-hexyl ITC substituted the methylsulfinyl group in 6-MSITC with formyl group, methylsulfinyl group, and methyl group, respectively.

Depending on the length of the alkyl chain of MSITC, there are a number of analogues of MSITC (Figure 3). 4-MSITC, also known as sulforaphane, is a major ITC of broccoli [71], and 2-MSITC and 8-MSITC are artificially synthesized [72]. The inhibitory potency on COX-2 and iNOS expression is increased depending on the alkyl chain elongation [11, 12], suggesting that the alkyl chain length is important for the inhibitory activity.

Noshita et al. [49] synthesized a series of ITCs (Figure 3) based on 6-MSITC to check the inhibitory activity against NO production in LPS-induced macrophages. Substitution of ITC group in 6-MSITC with thiocyanate group (1-(methylsulfinyl)-6-thiocyanatohexane, [49]) had no inhibitory effect, indicating the inhibitory action may entirely depend on its ITC group. The alkyl chain elongation in allyl ITCs also increases their lipophilicity (log *P* value), suggesting that the inhibitory potency of inflammatory factors by ITCs may be related to their log *P* values. Furthermore, substitution of the methylsulfinyl group in 6-MSITC with a formyl (6-isothiocyanatohexanal [49, 73]), a methylsulfanyl (1-isothiocyanato-6-(methylthio)hexane [49, 74]),  or a methyl (*n*-hexyl ITC [49]), attenuated the inhibitory activity. Polar surface area (PSA) value of these analogues is lower than that of 6-MSITC. In addition, the inhibitory potencies of ITCs showed better correlation with their PSA values rather than their log *P* values. Taken together, 6-MSITC has potent biological activity because of its higher PSA value and some degree of log *P* value [49]. 

## 6. Cellular Uptake of 6-MSITC

We investigated the effect of 6-MSITC on the binding of fluorescein-labeled LPS to the LPS receptor by a flow cytometry analysis, and the data suggested that 6-MSITC could not affect the binding of LPS to the receptor in plasma membrane in RAW264 cells (unpublished data). Thus, 6-MSITC has no influence on the interaction of LPS receptors. Several studies have revealed the metabolism of ITCs in several cell lines [4, 70, 75–77]. ITCs appear to penetrate cellular membrane by diffusion and rapidly conjugate with intracellular reduced glutathione (GSH) via their ITC group (–N=C=S). The methylsulfinyl group (CH_3_–S(=O)–) and the length of alkyl chain of 6-MSITC might contribute to the cell membrane permeability [49]. GSH is an important intracellular redox buffer that exits as a reduced predominant form, as a disulfide form (GSSG), or as mixed disulfide (GSSR) with protein thiols [78]. The redox status within the cells, reflected by GSH/GSSG [79], has been shown to be relevant for the regulation of inflammatory genes [80]. However, the detailed relationship between GSH-conjugated ITCs and signaling pathways involved in inflammatory factors is not clear. Future studies are needed to elucidate the role of GSH-conjugated 6-MSITC in LPS-induced cellular signaling pathways.

## 7. Conclusions

We have demonstrated that 6-MSITC has inhibited several inflammatory factors such as COX-2, iNOS, and inflammatory cytokines at the transcription factor/promoter levels. MAPK signaling pathways are one of the important pathways involved in inflammatory responses, and 6-MSITC suppresses all of three MAPK pathways leading to activation of transcriptional factors. Molecular analysis by MAPK inhibitors revealed the relationship between the transcriptional factors and MAPKs inhibited by 6-MSITC. 6-MSITC blocks LPS-induced COX-2 expression by suppressing ERK and p38 kinase signaling cascades leading to the activation of CREB and C/EBP*δ*, and by inhibiting JNK cascade leading to AP-1 activation. On the other hand, 6-MSITC attenuates iNOS expression mainly by blocking AP-1 activation. In addition, 6-MSITC inhibits JAK2 signaling pathway, which upregulates the expression of inflammatory factors through STAT phosphorylation, C/EBP*δ* expression, and JNK-mediated AP-1 activation. We also clarified the structure-activity relationship of MSITC analogues. 6-MSITC has potential usefulness as an anti-inflammatory agent because of its higher PSA value and some degree of log *P* value.

In recent years, numerous epidemiological and experimental animal studies have shown strong anti-inflammatory and chemopreventive effects of natural products. The elucidation of molecular mechanisms underlying the action of natural compounds may provide further insights into their potential usefulness as anti-inflammatory agents. The further studies on anti-inflammatory properties of 6-MSITC in clinical trial will greatly expand the development of 6-MSITC as an anti-inflammatory agent.
